# Multiple Roles of Myd88 in the Immune Response to the Plague F1-V Vaccine and in Protection against an Aerosol Challenge of *Yersinia pestis* CO92 in Mice

**DOI:** 10.1155/2014/341820

**Published:** 2014-06-04

**Authors:** Jennifer L. Dankmeyer, Randy L. Fast, Christopher K. Cote, Patricia L. Worsham, David Fritz, Diana Fisher, Steven J. Kern, Tod Merkel, Carsten J. Kirschning, Kei Amemiya

**Affiliations:** ^1^US Army Medical Research Institute of Infectious Diseases, Fort Detrick, Frederick, MD 21703, USA; ^2^Center of Biologics Evaluation and Research, US Food and Drug Administration, Bethesda, MD 20892, USA; ^3^Institute of Medical Microbiology, University Duisburg-Essen, Essen, Germany

## Abstract

The current candidate vaccine against *Yersinia pestis* infection consists of two subunit proteins: the capsule protein or F1 protein and the low calcium response V protein or V-antigen. Little is known of the recognition of the vaccine by the host's innate immune system and how it affects the acquired immune response to the vaccine. Thus, we vaccinated Toll-like receptor (Tlr) *2*, *4*, and *2/4*-double deficient, as well as signal adaptor protein *Myd88*-deficient mice. We found that Tlr4 and Myd88 appeared to be required for an optimal immune response to the F1-V vaccine but not Tlr2 when compared to wild-type mice. However, there was a difference between the requirement for Tlr4 and MyD88 in vaccinated animals. When F1-V vaccinated *Tlr4* mutant (lipopolysaccharide tolerant) and *Myd88*-deficient mice were challenged by aerosol with *Y. pestis* CO92, all but one *Tlr4* mutant mice survived the challenge, but no vaccinated *Myd88*-deficient mice survived the challenge. Spleens from these latter nonsurviving mice showed that *Y. pestis* was not cleared from the infected mice. Our results suggest that MyD88 appears to be important for both an optimal immune response to F1-V and in protection against a lethal challenge of *Y. pestis* CO92 in F1-V vaccinated mice.

## 1. Introduction

The first vaccine developed against plague was a heat-inactivated, whole-cell vaccine used by Haffkine during the Third Pandemic of plague in India in 1897 [1]. For the next 100 years, heat-inactivated, formalin-inactivated, or live-attenuated whole-cell vaccines were used to vaccinate humans against plague infection. The current candidate plague vaccine consists of a F1 capsule protein and the low calcium response (Lcr) V protein or V-antigen either as a mixture of the two proteins or a recombinant fusion of the two proteins [2, 3].

A strong humoral immune response to the individual subunits F1 or V or combined subunits (F1-V or F1+V), or an altered V-antigen (V10) was initially believed to be sufficient to provide protection against a lethal* Y. pestis* challenge in both mouse and nonhuman primate models of plague [2–8]. Both murine and human monoclonal antibodies against the subunit components of the plague vaccine have been shown to mediate protection against a lethal plague challenge in mice [9–12].

There is evidence to suggest that cell mediated immune responses are also important for protection against* Y. pestis* infection [13–15]. Although there are still some questions as to the contribution of the humoral and cellular immune responses for protection mediated by the plague vaccine in animal models, the F1-V subunit vaccine is currently being evaluated in a human Phase 2b clinical trial [16]. Very little is known of the host's innate immune response to the F1-V vaccine, and its effect on the ability of the vaccinated host to be protected from a lethal aerosol challenge by* Y. pestis* CO92. Thus we wanted to evaluate the involvement of Tlr2, Tlr4, and MyD88 in raising antibodies to the F1-V subunit vaccine, and then determine if vaccinated mice with specific deficiencies in these Tlrs or adaptor protein were protected in an aerosol challenge model with the virulent* Y. pestis* CO92 strain.

## 2. Materials and Methods

### 2.1. Reagents

The F1-V and V-antigen preparations were obtained from Dr. Brad Powell (USAMRIID, Ft. Detrick, MD). F1-V was prepared as previously described [17], and F1- and V-antigens were prepared as described by Heath et al. [18]. Endotoxin was removed from F1-V and V-antigens by Dr. Bill Gillette at the National Cancer Institute (NCI) (Frederick, Maryland). F1-V preparations contained endotoxin levels < 0.2 EU/*μ*g as determined by Lonza (Walkersville, MD) using the kinetic chromogenic* Limulus* amebocyte lysate method. Anti-F1 monoclonal antibody (clone F1-04-A-G1) for immunohistochemical analysis was obtained from the USAMRIID cell culture division.

### 2.2. Animal Experiments

#### 2.2.1. Mice

The original* Tlr2 deficiency* was in C57BL/6 mice which was a kind gift from Tularik (South San Francisco, CA), and backcrossed to C3H/HeJ.* Tlr2*,* Tlr4*, and* Tlr2*/*Tlr4* deficient C3H female mice were approximately 14 weeks old and backcrossed to C3H/HeN wild-type mice 9 times [19, 20]. In the first* Myd88* deficient vaccine study male C57BL/6 mice approximately 10 weeks old were used and were aged matched with control female C57BL/6 mice that were obtained from the NCI, Frederick, MD. The* Myd88* deficient mice were a kind gift from Dr. Shizuo Akira [21] and were backcrossed to a C57BL/6J background for over eight generations [22]. C57BL/6* MyD88* deficient female mice used in the challenge study were approximately 6–10 weeks old. Sex and aged-matched C57BL/6 mice were obtained from NCI, Frederick, MD. C3H/HeJ [lipopolysaccharide (LPS) tolerant] 6–8 weeks old female mice (hereafter referred to as* Tlr4* mutant) were used for the* Tlr4* mutant challenge studies, and age and sex matched C3H/HeN control mice were obtained from NCI, Frederick, MD [23–25].

Research was conducted under an IACUC approved protocol in compliance with the Animal Welfare Act, PHS Policy, and other federal statutes and regulations relating to animals and experiments involving animals. The facility where this research was conducted is accredited by the Association for Assessment and Accreditation of Laboratory Animal Care, International and adheres to the principles stated in the 8th Edition of the Guide for the Care and Use of Laboratory Animals, National Research Council, 2011.

#### 2.2.2. Vaccinations

Mice were vaccinated with F1-V twice subcutaneously as described previously [26] with Alhydrogel (500 ug) (Brenntag Biosector, Denmark). The amount of F1-V used is described in the tables or figure legends. Mice received either adjuvant or adjuvant with indicated amounts of F1-V. Serum was obtained from mice by intracardiac puncture or retroorbital bleeding approximately 4 weeks after the initial vaccination or 3-4 weeks after the boost vaccination. Mice were challenged by aerosol 22–30 days after the boost vaccination with* Y. pestis* CO92 where 1 LD_50_ is 6.8 × 10^4^ colony forming units (cfus) for a whole body challenge with amounts specified in the figure legend [27]. Mice were observed for 21 days postchallenge.

### 2.3. Antibody, Cytokine, and Proliferation Assays 

#### 2.3.1. Antibody Titers

Antibody titers against the vaccine were determined by enzyme-linked immunosorbent assay (ELISA) as described previously [26] approximately 30 days after the initial vaccination and 22–30 days after the boost vaccination. All antibody titers were performed in triplicate for each mouse and reported as the geometric mean with standard error of the mean (SEM).

#### 2.3.2. Cytokine Analysis

Cytokines expressed by stimulated splenocytes were determined as previously described [26]. Briefly, spleen cultures were prepared from mice approximately 30 days after the last vaccination. Spleens were combined in pairs within each group and duplicate cultures (5×10^6^ cells/mL) were prepared and stimulated with F1-V (10 *μ*g/mL) or V-antigen (10 *μ*g/mL) for 40–45 h at 37 C with 5% CO_2_. The culture supernatants were collected, and the amount of cytokine expression (in triplicate) was determined by BD FACSArray analysis (BD Pharmingen, San Diego, CA). The limits of detection were as follows: IFN-*γ*, 0.5 pg/mL; IL-12 (p70), 1.9 pg/mL; IL-4, 0.3 pg/mL; and IL-10, 9.6 pg/mL. The results were reported as the mean with the standard error of the mean.

#### 2.3.3. Proliferation Analysis

Proliferation of splenocytes was determined as previously described [26]. Briefly, splenocyte cultures (0.2 mL) were prepared from spleens as previously described above except at 2 × 10^6^ cells/mL and incubated with the antigen in triplicate for 40–45 h at 37 C with 5% CO2. Incubation was continued for 18–24 h with 1 *μ*Ci of [3H] thymidine at a specific activity of 5 Ci/mmol (Amersham Life Sciences, Arlington, IL) before collecting the cells and counting the amount of radioactivity incorporated. The results were reported as the mean with the standard error of the mean.

### 2.4. Histochemical/Immunohistochemical Analysis of Spleens

Spleens of mice in the* Myd88*-deficient vaccine study were removed as soon as we found the nonsurviving mouse early in the morning or through the work day up to 21 days after challenge. Mice that survived the challenge after 21 days or mice that were unchallenged control mice were deeply anesthetized and euthanized before removing their spleens. All spleens were placed into 4% buffered formalin for at least 21 days before histochemical or immunohistochemicial analysis was performed by the Pathology Division at USAMRIID. Tissue sections were stained using an automated hematoxylin and eosin Sakura Automated slide stainer. Immunohistochemical staining was performed using a Dako EnVision+ kit (Dako, Catalog no. K4007 or K4010) with the* Y. pestis* anticapsule F1 monoclonal antibody as the primary antibody. All photomicrographs were taken with a Nikon Eclipse 80i microscope using a 20X objective lens with the final magnification of 271X. The histological slides were read by the Veterinarian pathologist in the Bacteriology Division at USAMRIID.

### 2.5. Statistical Analysis

Log 10 transformations were applied to antibody titer values and cytokine responses before analysis to satisfy assumptions of normality and homoscedasticity. Antibody titers were compared between experimental groups using two-sample *t*-tests when comparing only two groups or Dunnett's tests when comparing multiple groups to a shared control. Mean times to death were compared with two-sample *t*-tests with stepdown Bonferroni corrections to account for multiple comparisons. Survival rates were compared with Fisher's exact tests with stepdown Bonferroni corrections to account for multiple comparisons. All statistical analyses were conducted using SAS Version 9 (SAS Institute Inc., Cary, NC, 2003). All hypothesis tests are two-sided and considered significant at the *α* = 0.05 level.

## 3. Results

### 3.1. Antibody Response, Cytokine Expression, and Proliferative Response to the F1-V Vaccine

**Figure 1:**
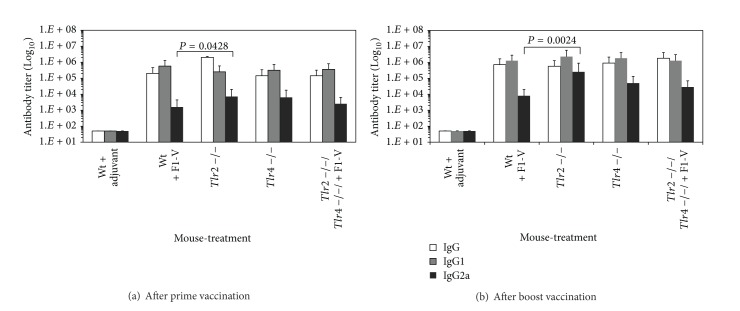
Comparison of the class and subclass antibody titers to the* Y. pestis* F1-V vaccine in wild-type C3H/NeN,* Tlr2*,* Tlr4*, and* Tlr2/4* deficient mice after the prime and boost vaccination. Blood was drawn 30 days after each vaccination for the antibody determination (IgG, IgG1, and IgG2a) that is reported as the geometric mean with geometric standard error of the mean. There were 6 mice in each group. There was a significant difference between the wild-type C3H/HeN mice and* Tlr2* deficient C3H/HeN mice in the levels of IgG2a after the prime vaccination (*P* = 0.0428) and boost vaccination (*P* = 0.0024).

C3H/HeN mice with deficiencies in* Tlr2*,* Tlr4*, or both* Tlr2/4* were vaccinated twice with 1 *μ*g of F1-V, and antibody titers determined 30 days after the prime and boost vaccinations.  Figure 1 shows that 30 days after the first vaccination, there was not a significant difference in the levels of IgG between the wild-type and* Tlr2*,* Tlr4*, or* Tlr2/4* double deficient mice (*P* = 1.000). There was also little difference in the IgG1 and IgG2a subclass response to the vaccine in the same mice, except in one case there was a slight significant difference in the IgG1 response between the wild-type mice that received the vaccine and mice with the* Tlr2* deficiency (*P* = 0.042). The IgG or subclass response to the vaccine was also not in most cases significantly different 30 days after the boost vaccination (*P* = 1.000) between the wild-type C3H/HeN mice,* Tlr2*,* Tlr4*, and* Tlr2/4* double deficient mice (Figure 1). There was a significant difference between the IgG2a response between the wild-type mice and the* Tlr2* deficient mice (*P* = 0.0024). Nevertheless, our results suggest that overall, the antibody response to the plague F1-V vaccine did not depend on the presence of Tlr2 or Tlr4.

**Figure 2:**
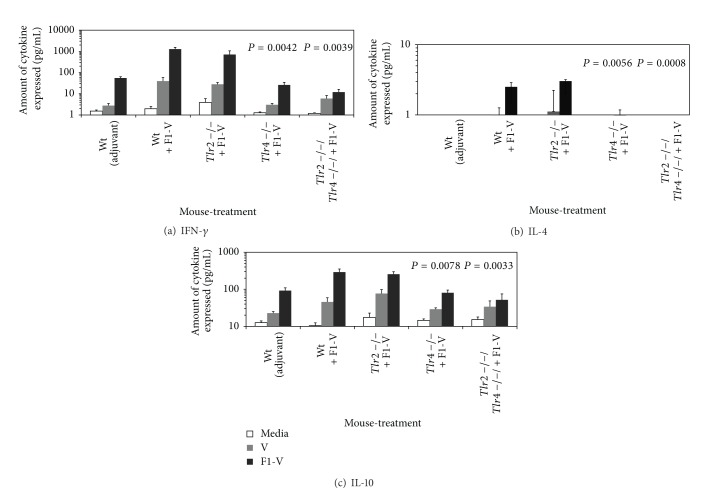
Cytokine expression by stimulated splenocytes from F1-V vaccinated wild-type C3H/HeN,* Tlr2*,* Tlr4*, and* Tlr2/4* deficient mice appears to be dependent on* Tlr4*. Mice were given a prime-boost vaccination of F1-V (1 *μ*g) as shown in Figure 1, and splenocytes from these same mice were prepared and stimulated for 44 h in triplicate with medium only, 4 *μ*g of F1-V or V antigen. Culture supernatants were collected and cytokine expression was determined: (a) IFN-*γ*; (b) IL-4; (c) IL-10. The results are reported as mean with standard error of the mean. Statistical significance shown above the respective bar was reported for differences in cytokine expression between splenocytes from wild-type C3H/HeN and* Tlr4 *and* Tlr2/4*-deficient C3H/HeN mice stimulated with F1-V.

We then evaluated cytokine and proliferative responses to the vaccine by splenocytes from F1-V vaccinated wild-type,* Tlr2*,* Tlr4*, and* Tlr2/4* double deficient mice. Although we did not vaccinate these mice with only V-antigen, we wanted to compare its ability to stimulate splenocytes like the F1-V vaccine. We saw little IFN-*γ*, which is a T-cell helper type 1 (Th1-) like cytokine, produced by splenocytes from wild-type mice that received only adjuvant (Figure 2(a)). Splenocytes from wild-type C3H/HeN mice responded well to F1-V producing the most IFN-*γ* (1272 pg/mL), but splenocytes from* Tlr2* deficient mice produced a little more than half the amount of IFN-*γ* (710 pg/mL) as the splenocytes from wild-type C3H/HeN mice (*P* = 0.2050). Neither splenocyte preparations produced very much IFN-*γ* in response to the V-antigen. Splenocytes from F1-V vaccinated* Tlr4 *deficient or* Tlr2/4* double deficient mice produced little IFN-*γ* (26.2 and 12.1  pg/mL, resp.) in response to F1-V (*P* = 0.0042 or *P* = 0.0039, resp.). We also examined the expression of IL-12 (p70) but very little was detected in all splenocyte preparations after stimulation with F1-V (data not shown).

We examined the expression of two Th2-type cytokines, IL-4 and IL-10. No IL-4 was detected in splenocyte cultures from mice that received only the adjuvant in response to V-antigen or F1-V (Figure 2(b)). Very low amounts of IL-4 were detected in splenocytes from wild-type C3H/HeN,* Tlr2* and* Tlr4* deficient mice (2.51, 3.03, and 0.59 pg/mL, resp.). No IL-4 was detected in splenocyte cultures from* Tlr2*/*Tlr4* double deficient mice after stimulation with the vaccine. There were significant differences in the amount of IL-4 produced by splenocytes from* Tlr4* and* Tlr2/4* deficient mice compared to splenocytes from the wild-type C3H/HeN mice (*P* = 0.0056 and *P* = 0.0008, resp.). Splenocytes from F1-V vaccinated wild-type C3H/HeN or* Tlr2* deficient mice produced comparable amounts of IL-10 (290 and 255 pg/mL, resp.) when stimulated with F1-V (*P* = 0.9023), but not with the V-antigen (Figure 2(c)). Significantly less IL-10 was expressed by splenocytes from F1-V vaccinated* Tlr4* and* Tlr2/4* deficient mice (80.5 and 51.9 pg/mL, resp.) when stimulated by the vaccine (*P* = 0.0078 and *P* = 0.0033, resp.). Little IL-10 was induced by V-antigen compared to that by F1-V at the same time.

**Figure 3:**
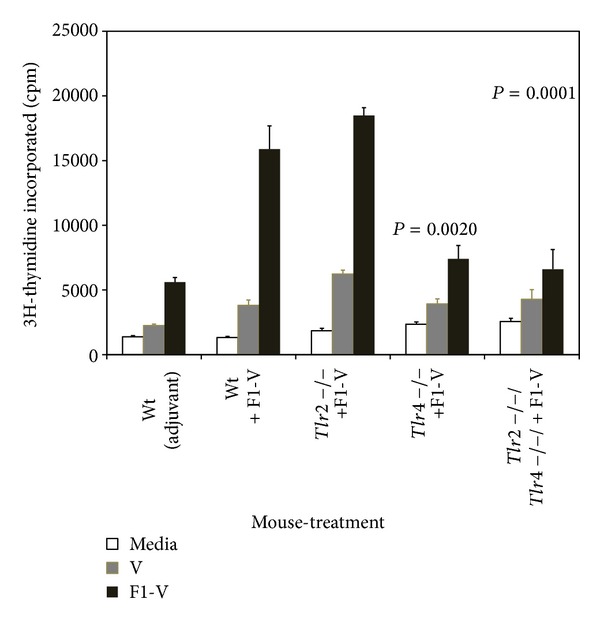
Proliferation by stimulated splenocytes from F1-V vaccinated wild-type C3H/HeN,* Tlr2*,* Tlr4*, and* Tlr2/4*-deficient mice appears to be dependent on Tlr4. Proliferation was determined after 44 h incubation in the presence of 4 *μ*g of F1-V or V-antigen and a further 24 h incubation in the presence of 3[H]-Thymidine. Splenocytes used in the assay were prepared from the same groups of mice as shown in Figure 1. The results are reported as mean with standard error of the mean. The results on the *y*-axis are plotted in linear because of the overall low amount of stimulation. Statistical significance shown above the respective bar was reported for differences between the amount of proliferation between splenocytes from wild-type C3H/HeN and* Tlr2* and* Tlr2/4*-deficient C3H/HeN mice stimulated with F1-V.

The proliferative response to the F1-V vaccine and V-antigen was examined (Figure 3). When the amount of proliferation by splenocytes in response to the V-antigen alone between the wild-type C3H/HeN,* Tlr2*,* Tlr4*, and* Tlr2/4* deficient mice was compared, we saw a significant increase in proliferation by the splenocytes from the* Tlr2* deficient mice (*P* = 0.0059) but not by the other splenocytes. Splenocytes from wild-type C3H/HeN and* Tlr2* deficient mice that received the vaccine, proliferated well (12- to 10.5-fold, resp.) in response to the vaccine but with no significant difference between the two strains of mice (*P* = 0.7013). In contrast, there was a significant decrease in the proliferative response to the vaccine by splenocytes from* Tlr4 *and* Tlr2/4* double deficient mice (*P* = 0.0020 and *P* = 0.0001, resp.). These results suggest that cellular immune responses to F1-V were more dependent on the presence of Tlr4 but not Tlr2 in mice vaccinated with F1-V.

### 3.2. Antibody Response, Cytokine Expression, and Proliferative Response by* Myd88* Deficient Mice

**Figure 4:**
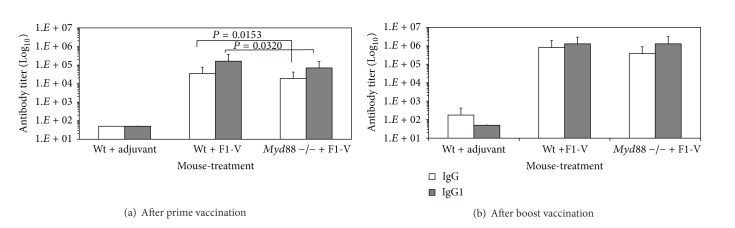
Antibody response to the plague F1-V vaccine in wild-type C57BL/6 and* Myd88* deficient mice after the prime and boost vaccination. Serum for the prime vaccination was drawn 22 days after vaccination and for the boost vaccination 29 days after vaccination. All mice received 2 *μ*g of F1-V except mice in the adjuvant only group. N for wild-type C57BL/6 mice with adjuvant and wild-type C57BL/6 mice with F1-V was 6, while for the* Myd88* deficient C57BL/6 group with F1-V was 9. The titers are reported as geometric mean with geometric standard error of the mean. Significant differences in the antibody titer between the wild-type C57BL/6 mice that received F1-V and* Myd88*-deficient C57BL/6 mice that received F1-V which is shown above the respective bar after the prime vaccination (a).

Because the MyD88 adaptor protein is a pivotal molecule in the innate immune response, we wanted to examine the contribution of MyD88 to the antibody and cellular immune responses to the vaccine. We vaccinated 3 groups of mice with or without F1-V (Figure 4). After the initial vaccination, we saw significant differences in the IgG and IgG1 levels between the wild-type C57BL/6 mice and* Myd88* deficient mice (*P* = 0.0153 and *P* = 0.0320, resp.) 22 days later. However, after the boost vaccination, there was a difference but not significant (*P* = 0.1161) between the IgG levels of the wild-type C57BL/6 and* Myd88* deficient mice. There was no difference in the IgG1 response between the wild-type C57BL/6 and* Myd88* deficient mice. We saw little IgG2a produced in response to the vaccine (data not shown) because C57BL/6 mice do not have a functional IgG2a gene but produce a different isotype [IgG2c, 28-31].

**Figure 5:**
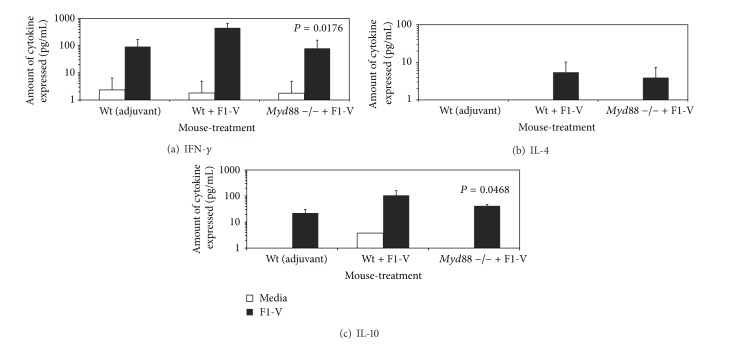
Cytokine expression by stimulated splenocytes from F1-V vaccinated wild-type C57BL/6 or* MyD88*-deficient mice appears to require MyD88. Splenocytes were stimulated with F1-V (5 *μ*g) or medium alone for approximately 45 h before collecting the supernatant and determining the amount of cytokine present: (a), IFN-*γ*; (b), IL-4; (c), IL-10. Cells from three different groups of mice were used (see Figure 4): (1) wild-type (Wt) C57BL/6 mice that received only adjuvant, (2) Wt C57BL/6 mice that received F1-V (2 *μ*g), and (3)* Myd88*-deficient C57BL/6 mice that received F1-V (2 *μ*g). The results are reported as mean with standard error of the mean. Statistical significance shown above the respective bar was reported for differences between the amount of cytokine expressed between the wild-type C58BL/6 and* Myd88*-deficient splenocytes stimulated with F1-V (*P* = 0.0176 and *P* = 0.0468) in panels (a) and (c), respectively.

We then examined the expression of IFN-*γ*, IL-4, and IL-10 by splenocytes cultures from F1-V vaccinated wild-type and* Myd88* deficient mice (Figure 5). IFN-*γ* (451 pg/mL) and IL-10 (106 pg/mL) were expressed in moderate amounts by splenocytes from F1-V vaccinated mice but not IL-4 (6.84 pg/mL). We saw significantly lower amounts of expression of IFN-*γ* (79.7 pg/mL, *P* = 0.0176) and IL-10 (42.04 pg/mL, *P* = 0.0468) but not IL-4 (3.92 pg/mL, *P* = 0.0728) by splenocytes from* Myd88* deficient mice. We also detected IL-12 (p70) produced in very low amounts (7.82 pg/mL) by vaccine stimulated splenocytes from wild-type C57Bl/6 mice but not from splenocytes from* Myd88* deficient mice (data not shown).

**Figure 6:**
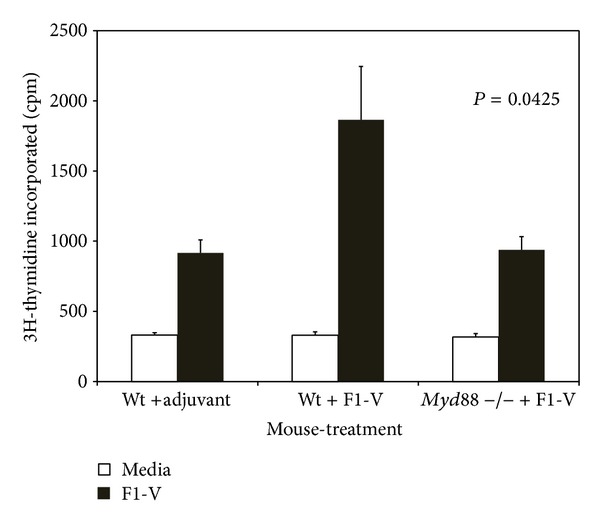
Proliferation by stimulated splenocytes from F1-V vaccinated wild-type C57BL/6 mice or* Myd88*-deficient C57BL/6 mice requires MyD88. Proliferation of stimulated splenocytes was determined as in Figure 3 except 2 *μ*g of F1-V was used for stimulation in 0.25 mL. The same source of splenocytes stated in Figure 5 was used. The results are reported as mean with the standard error of the mean. Statistical significance shown above the respective bars was reported for differences between the amount of proliferation between splenocytes from wild-type C57BL/6 and* Myd88*-deficient C57BL/6 mice stimulated with F1-V.

We compared the proliferative response of splenocytes from wild-type and* Myd88* deficient mice vaccinated with the plague vaccine (Figure 6) and saw close to a twofold decrease in proliferation between these two groups (*P* = 0.0425). Our results suggest that MyD88 protein appears to be intricately involved in the immune response to the F1-V vaccine.

### 3.3. MyD88 but Not Tlr4 Is Required for Survival against* Y. pestis* in F1-V Vaccinated Mice

**Figure 7:**
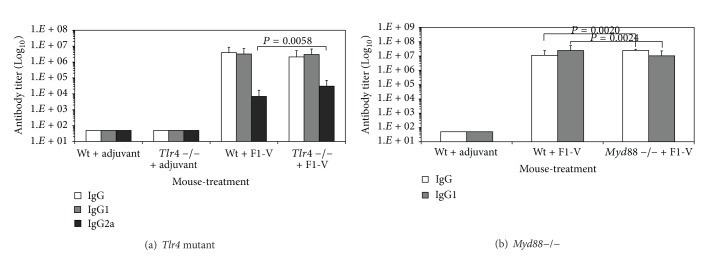
Antibody titers against the F1-V vaccine in (a) C3H/HeN wild-type and* Tlr4* mutant C3H/HeJ mice, and (b) C57BL/6 wild-type and* Myd88*-deficient C57BL/6 mice vaccinated with F1-V before* Y. pestis* CO92 aerosol challenge. (a) There were 4 groups of mice with 10 in each group: (1) Wt+adjuvant, (2)* Tlr4* mutant+adjuvant, (3) Wt+F1-V (2.9 *μ*g), and (4)* Tlr4* mutant+F1-V (2.9 *μ*g). Antibody titers are reported from serum collected 22 days after a boost vaccination. (b) There were 3 groups of mice with 15 in groups 1 and 2 and 14 in group 3:  (1) Wt+adjuvant, (2) Wt+F1-V (2.5 *μ*g), (3)* Myd88*-deficient mice+F1-V (2.5 *μ*g). Antibody titers are reported as geometric mean with geometric standard error of the mean. Significant differences between the antibody class or subclass are reported above the respective bar between the (a) Wt+F1-V and* Tlr4* mutant+F1-V or (b) Wt+F1-V and* Myd88*-deficient+F1-V.

**Figure 8:**
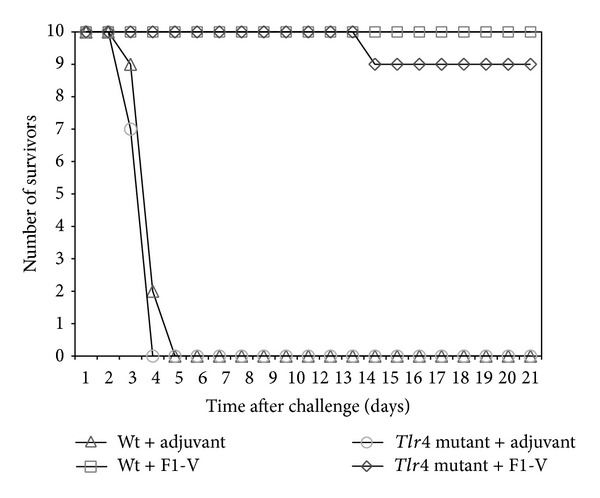
Survival of F1-V vaccinated C3H/HeN wild-type and C3H/HeJ* Tlr4* mutant mice after aerosol challenge with* Y. pestis* CO92. Mice were given two vaccinations of F1-V (2.9 *μ*g), and 22 days after the boost vaccination, they were challenged by aerosol with 21 LD_50_ of* Y. pestis* CO92. There were four groups of mice (10 mice per group): wild-type C3H/HeN, with adjuvant only (∆);* Tlr4* C3H/HeJ mutant, with adjuvant only (○); wild-type C3H/HeN, with F1-V (□); and* Tlr4* C3H/HeJ mutant, with F1-V (◊). After challenge, the mice were followed for 21 days. Previous results were essentially the same when the mice in the same vaccination groups were challenged with half the dose (10 LD_50_) of* Y. pestis* CO92 except all (10/10) the F1-V vaccinated C3H/HeJ* Tlr4* mutant mice survived.

It appeared that cell-mediated immune responses in F1-V vaccinated* Tlr4* and* Myd88* deficient mice were affected more than in* Tlr2* deficient mice. It had been previously suggested that weak or inefficient activation of Tlr2 by LcrV makes it unlikely that Tlr2 is involved in pathogenesis by plague [28, 29]. We, therefore, examined the contribution of Tlr4 and MyD88 in protection against an aerosol challenge by* Y. pestis* CO92 after F1-V vaccination. We used C3H/HeJ mice that have a missense mutation in the* Tlr4* coding region that makes it unresponsive to LPS [23–25] and C3H/HeN mice for the wild-type* Tlr4* (Figure 7(a)). One group of* Tlr4* mutant C3H/HeJ mice received adjuvant and another group received both adjuvant and F1-V (2.9 *μ*g). Another group of wild-type C3H/HeN mice received only adjuvant and another group received adjuvant with F1-V. Before challenge there was a lower IgG response to the vaccine by the* Tlr4* mutant mice, but the difference in either IgG or IgG1 titers to the vaccine between the wild-type C3H/HeN and* Tlr4* mutant C3H/HeJ mice was not significant (1.87-fold and 1.07-fold, resp.). There was also no significant difference in the distribution of IgG levels against the F1- or V-antigens between the strains before challenge (data not shown). Twenty-two days after the boost vaccination all groups of mice were challenged with 21 LD_50_ of* Y. pestis* CO92 by aerosol.  Figure 8 shows that only one F1-V vaccinated* Tlr4* mutant C3H/HeJ mouse died from the challenge, while no wild-type C3H/HeN vaccinated mice were lost. By comparison, C3H/HeN and C3H/HeJ mice that received only adjuvant all died by day 4-5 postchallenge. This study was repeated previously, with the same groups of vaccinated mice (*n* = 10 for all groups), except they were challenged with a lower dose (10 LD_50_) of* Y. pestis* CO92. In this case there was complete protection of F1-V vaccinated* Tlr4* mutant mice (10/10) and no protection of the C3H/HeN and C3H/HeJ mice that received only adjuvant as in the study with the higher challenge dose (data not shown). Our results suggest that in F1-V vaccinated mice Tlr4 does not contribute significantly towards an antibody response to the vaccine or toward protection against a lethal aerosol challenge by* Y. pestis* CO92.

**Figure 9:**
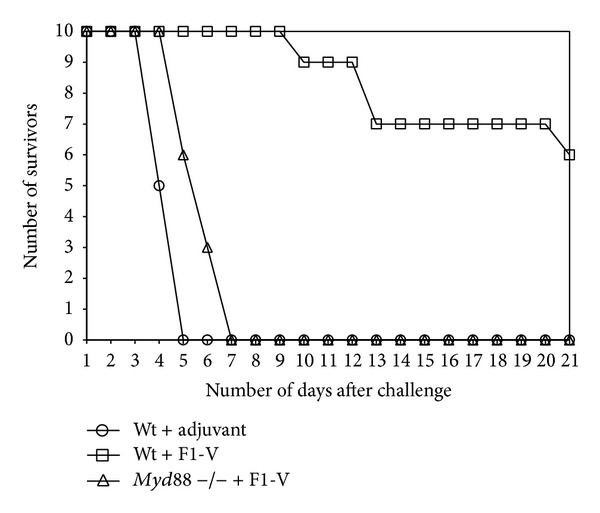
Survival of F1-V vaccinated C57BL/6 wild-type and C57BL/6* Myd88*-deficient mice after aerosol challenge with* Y. pestis* CO92. Mice were given two vaccinations of F1-V (2.5 *μ*g) (see Figure 7(b)), and 33 days after the boost vaccination they were challenged by aerosol with 19 LD_50_ of* Y. pestis* CO92. There were three groups of mice that were challenged (10 mice per group): wild-type C57BL/6+adjuvant only (○); wild-type C57BL/6+F1-V (□); and C57BL/6* Myd88*-deficient mice+ F1-V (∆). After challenge, the mice were followed for 21 days. The mean time to death (MTD) was the following: wild-type with adjuvant only: 4.50 days; wild-type with F1-V: 14.25 days;* Myd88*-deficient mice with F1-V: 5.90 days.

To examine the role of MyD88 in protection against a* Y. pestis* challenge in F1-V vaccinated mice, we vaccinated 3 groups of mice (Figure 7(b)). The first group consisted of wild-type C57BL/6 mice that received only adjuvant, and the second group of wild-type C57BL/6 mice received adjuvant with F1-V (2.5 *μ*g). The last group consisted of C57BL/6* Myd88* deficient mice that received adjuvant with 2.5 *μ*g of F1-V. Although the* Myd88* deficient F1-V vaccinated mice had a substantial IgG and IgG1 titer against the vaccine, there was a significantly lower antibody titer than in the wild-type mice (*P* = 0.0020 and *P* = 0.0024, resp.) against the vaccine and individual subunits (*P* = 0.0053 and *P* = 0.0338, resp., data not shown). When 10 mice from each group were challenged by aerosol with 10 LD_50_ of* Y. pestis *CO92, all mice that received only adjuvant died within 3–5 days after challenge, while F1-V vaccinated* Myd88* deficient mice all died within 5–7 days after challenge (Figure 9). There was no significant difference between the mean time to death (MTD) of the adjuvant only wild-type group and the F1-V vaccinated* Myd88* deficient group (*P* = 0.1151). In contrast, 6 out of 10 mice survived the challenge in the wild-type F1-V vaccinated mice. The MTD for these three groups of mice were 4.5 days, 5.9 days, and 14.3 days, respectively. There was a significant difference between the MTD between the F1-V vaccinated wild-type mice and F1-V vaccinated* Myd88* deficient mice (*P* < 0.0001) and in the survival rate (*P* = 0.0325). Unlike Tlr4, the MyD88 adaptor protein appears to be required for an optimal antibody response to the vaccine and for protection against* Y. pestis* in F1-V vaccinated mice.

### 3.4. Spleens from Nonsurviving Challenged F1-V Vaccinated* Myd88*-Deficient Mice Showed That the Pathogen Was Not Cleared

**Figure 10:**
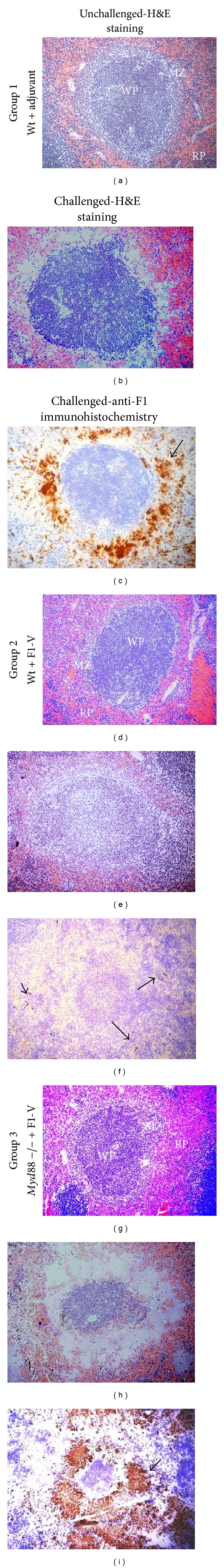
Histochemical and immunohistochemical analysis of spleens from unchallenged and challenged mice from adjuvant only C57BL/6 wild-type, F1-V vaccinated C57BL/6 wild-type, and F1-V vaccinated C57BL/6* Myd88*-deficient mice. Mice were obtained from the corresponding group of mice as described in Figures 7(b) and 9. Spleens from additional mice from each group were used as controls (Unchallenged-H&E, panels (a), (d), and (g)). Regions in the spleen were labeled: white pulp, WP; red pulp, RP; and marginal zone, MZ. Spleen sections from challenged mice are shown stained (Challenged-H&E, panels (b), (e), and (h)) or probed with an anti-F1 monoclonal antibody (Challenged-Anti-F1, panels (c), (f), and (i)). The spleen section shown from Group 2, wild-type+F1-V was from a mouse that survived challenge (panels (e) and (f)). Arrows in panel (c) and (i) point to F1 positive regions in the marginal zone. Arrows in panel (f ) point to isolated F1 positive spots.

Histochemical and immunohistochemical analyses were performed on spleens from mice from the* Myd88* deficient mice challenge study (Figure 10). There were no significant lesions noted in any of the spleens from the three groups of mice before challenge that includes the spleens from the* Myd88* deficient mice (Figures 10(a), 10(d), and 10(g)). In wild-type C57BL/6 mice that received only adjuvant and died after challenge (Figure 10, Gp1a-c), there was evidence of active infection (primarily of neutrophils and macrophages) and numerous bacilli in the marginal zone surrounding the white pulp. The presence of* Y. pestis* was confirmed with an anti-F1 monoclonal antibody (mAb) (Figure 10, Gp1c). There was a depletion of lymphocytes in this region compared to the wild-type mice that were not challenged (Figure 10, Gp1a).

In spleens of surviving wild-type C57BL/6 mice that were vaccinated with F1-V and challenged with* Y. pestis* CO92, there was an influx of lymphocytes in the white pulp and extramedullary hematopoiesis in the surrounding red pulp region. When the spleen sections were probed with the anti-F1 mAb, a few isolated anti-F1-positive spots were found in the marginal zone of the white pulp (Figure 10, Gp2f, arrows).

Spleens from* Myd88* deficient mice that were vaccinated with F1-V and challenged with* Y. pestis* CO92 appeared much like the wild-type mice that received only adjuvant. The presence of bacteria was seen in the marginal zone of the white pulp with mild to moderate lymphoid depletion observed in the white pulp, as well as mild extramedullary hematopoiesis in the red pulp (Figure 10, Gp3 h). A large amount of anti-F1 positive regions could be seen in the marginal zone of the white pulp and surrounding red pulp (Figure 10, Gp3i). Over all, the spleens from mice that did not survive the* Y. pestis* CO92 challenge from either wild-type mice that received only adjuvant or the* Myd88* deficient group that were vaccinated showed the presence of large amounts of the organism in the marginal zone of the white pulp as well as lymphoid depletion in the white pulp. The analysis of the spleens from F1-V vaccinated* Myd88* deficient mice suggested that MyD88 was required for clearance of the pathogen after a lethal challenge of* Y. pestis *CO92.

## 4. Discussion

The results of our studies with the* Tlr2*,* 4*, and* 2/4* double deficient or adaptor protein* Myd88* deficient mice suggest that Tlr4 and MyD88 appear to be important for an optimal antibody response to the subunit F1-V plague vaccine, but MyD88 also appears to be required for protection against a lethal* Y. pestis* CO92 challenge in F1-V vaccinated mice. Furthermore, cell-mediated immune responses to the vaccine appear to be more dependent on Tlr4 and MyD88 but not necessarily Tlr2. The expression of Th1- and Th2-like cytokines (IFN-*γ*, and IL-4, IL-10, resp.) and cell proliferation were moderately effected by the absence of Tlr2, but in the absence of Tlr4 or MyD88 the immune response to the vaccine was significantly affected. Still, there was distinction between the absence of Tlr4 or MyD88 in the immune response and protection against a* Y. pestis *CO92 challenge. We saw that in F1-V vaccinated mice, Tlr4 does not appear to be required for protection against a lethal challenge as long as antibodies to F1-V were present. In the absence of MyD88, however, the presence of a substantial level of antibody to the F1-V vaccine did not protect F1-V vaccinated mice from a lethal challenge of* Y*.* pestis*. Differences in the number of survivors between the wild-type C57BL/6 and* Myd88* deficient F1-V vaccinated mice might be attributed to both the difference in the antibody response to the vaccine (Figure 7(b), *P* = 0.0020) and differences in the cell-mediated response. Because of the critical role played by macrophages and neutrophils in the spread and control of* Y. pestis* in the infected host [30, 31], we cannot rule out if their involvement becomes limited in the clearance and survival of the host in the presence of a* Myd88*-deficiency at the same time that the host's immune system is being compromised [28]. Although we did not include a* Myd88* deficient group that received only adjuvant, we believe that like the wild-type C57BL/6 mice that received only adjuvant, this group would not survive the lethal challenge by* Y. pestis *CO92. Without any protective anti-F1-V antibody, we would anticipate that the number and rate of survival would be similar to the wild-type C57BL/6 mice that did not receive the vaccine. As a comparison, although Tlr4 is upstream from MyD88 but the signal transduction pathway would still not be activated, in the previous challenge study with* Tlr4* mutants, the number of survivors and MTD of* Tlr4* mutant mice that did not receive F1-V was similar to the wild-type mice that did not receive the vaccine (between 4-5 days). Furthermore, there was no significant difference in the number of survivors and the survival rate of the* Myd88* deficient mice that did receive F1-V and the wild-type C57BL/6 group. We saw that in the absence of MyD88,* Y. pestis* accumulates in the marginal zone of the spleen in mice that succumb to the lethal challenge, but they did not accumulate in spleens of F1-V vaccinated wild-type C57BL/6 mice, although we could see a few isolated organisms in the latter case. It is not clear at this time how much of the accumulation of* Y. pestis* in the spleen could be attributed to the possible attenuation of neutrophils in the absence of MyD88 [32, 33] or other biological events in the infected host (see discussion further below).

Although it could be said that part of our observation on the requirement of Tlr4 or MyD88 on the immune response to the F1-V vaccine could be attributed to the presence of endotoxin in our vaccine preparations, there were very minor amounts in our vaccine preparations. In the initial* Tlr2* and* 4* deficient mouse immune studies, each mouse received 1 *μ*g of F1-V (Figure 1), which contained approximately 0.18 EU per vaccination (see Section 2). This amount of endotoxin is approximately equivalent to 0.018 ng according to a reported estimated value of 1 EU/mL which is approximately 0.1 ng/mL of endotoxin (Pierce LAL chromogenic endotoxin quantitation kit, Thermo Scientific, Rockford, IL). This amount of estimated endotoxin did not have a significant effect on the antibody response between the wild-type C3H/HeN and* Tlr2* or* Tlr4* deficient mice to the vaccine (Figure 1). At the same time, in the initial* Myd88* deficient study, each mouse received 2 *μ*g of F1-V (Figure 4) or 0.36 EU per vaccination, which was estimated to be 0.036 ng per mouse. We saw a lower but not significant difference in the IgG response to the vaccine in the* Myd88* deficient mice when compared to the wild-type C57BL/6 mice (*P* = 0.1161). For the* Tlr4* mutant and* Myd88*-deficient challenge studies, we used a different F1-V preparation (Figures 7(a) and 7(b), resp.). Excluding the adjuvant control mice, each mouse in the* Tlr4* mutant challenge study received 2.9 *μ*g of F1-V or an estimated amount of 0.0087 EU, and in the* Myd88* deficient challenge study, each test mouse received 2.5 *μ*g of F1-V or 0.0075 EU, which corresponds to approximately 0.00087 ng and 0.00075 ng of endotoxin for the* Tlr4* mutant mice and* Myd88* deficient mice, respectively. For the antibody response to the vaccine in the* Tlr4* mutant C3H/HeJ mice, it was lower than the wild-type C3H/HeN mice but not significantly (*P* = 0.1486). In the case of the* Myd88* deficient mice that were challenged, the* Myd88* deficient mice did have a significantly lower but still a substantial antibody response to the vaccine when compared to the wild-type C57BL/6 mice (*P* = 0.0020). The differences in the antibody response and cell-mediated immune response to the vaccine between the wild-type mice and corresponding* Tlr4 *mutant or* Myd88* deficient mice suggest that MyD88 may be more important for an optimal immune response to the F1-V vaccine than Tlr4.

Numerous reports suggest that MyD88, Tlr2, or Tlr4, is required for clearance or protection against a bacterial pathogen [34–46]. Our report is the first to describe that MyD88 is required even in the presence of a substantial level of F1-V specific antibody for protection against* Y. pestis *infection. That MyD88 may be involved in activities other than as a signal adaptor protein cannot be completely excluded. Blander and Medzhitov [47] reported that uptake of* Escherichia coli* through phagosome/lysosome fusion was slower in bone marrow derived macrophages (BMDM) from* Tlr2/4* double deficient and* Myd88* deficient mice compared to BMDM from wild-type mice. Yates and Russell [48] reported a significant decrease in phagosome/lysosome fusion in BMDM from* Myd88* deficient mice after particle internalization was independent of Tlr2 or Tlr4. Sun and Ding [49] demonstrated that MyD88 adaptor protein increased the half-life of IFN-*γ*-induced mRNA for both TNF-*α* and IP-10. Stabilization of the mRNA was dependent on the activation of p38 and the presence of adenine-uridine-rich elements in the 3′-untranslated region of the mRNA. A physical association between IFN-*γ* R1 and MyD88 was noted. These reports suggest that MyD88 plays a pivotal role in the innate immune process as an adaptor protein, in phagosome/lysosome fusion after pathogen internalization, and cell-mediated immune events that affect the response to the pathogen.

The F1-V subunit vaccine has been formulated with aluminum hydroxide in animal and human studies [2, 8, 16, 18, 26, 50, 51]. It has been proposed that activation of the immune response by aluminum hydroxide adjuvants occurs through a protein complex called the inflammasome [52]. Activation of the inflammasome leads to activation of caspase-1 resulting in cleavage of pro-IL-1B and pro-IL-18 to mature molecules that are excreted [53–56]. However, there are conflicting reports on the dependency of the inflammasome on specific antibody responses [54–59]. Furthermore, there are conflicting reports on the requirement for Tlr activation in general for antigen specific antibody responses [60, 61]. It is not clear if inflammasome activation can possibly replace Tlr activation for antigen recognition for specific antibody responses, but equally important is the potential roles of MyD88 in the immune response for protection and clearance of the pathogen from the infected host as we have shown in the present study.

## 5. Conclusion

We have shown by using mice with deficiencies in specific components of the innate immune system that the antibody response to the plague F1-V vaccine could be affected, but we still observed a substantial antibody response in most cases in the absence of these components. An optimal immune response to F1-V appears to require the presence of Tlr4 or MyD88 but not Tlr2. In addition, the antibody response to the vaccine in the absence of Tlr4 still protected the mouse from a lethal challenge by* Y. pestis* CO92, but it did not in the absence of MyD88. Further, it may be that part of the reason for the lack of protection against plague in* Myd88* deficient mice was possibly a combination of a suboptimal antibody response to the vaccine and attenuated cell-mediated immune responses that led to the inability to clear the pathogen from the mouse. The latter possibility may also include attenuation of macrophage or neutrophil recruitment or phagocytosis of the pathogen. MyD88 appears to be involved in multiple aspects of the immune response to the plague vaccine and protection against plague infection.
